# The Bones of the Hand

**Published:** 1888-08-11

**Authors:** 


### Physiology and its Uses.

### THE BONES OF THE HAND.

Of all the parts of a well-articulated skeleton of the human body, that which most excites the admiration of the ordinary student is the hand. The foot, to some extent, divides the honours with its rival; but it is less mobile and much less versatile in function, and must undoubtedly take the second place. The head of course, if we consider its chief use as the brain-case, will naturally take precedence in real importance; but as an object of contemplation it rather excites horror in the ordinary observer than admiration. Except for the movements of the lower jaw it is absolutely rigid; and this one solitary movement of the bony human head may be horrid or ludicrous, according to the disposition of the observer; but it seldom excites enthusiasm.

The most casual student of physical science cannot but admire the structure and functions of the hand. As a weapon of defence the doubled fist makes a most effective club-head, of which the arm is the shaft. A blow with the hand straight from the shoulder of a very strong man will knock down a bullock, and has often been known to kill a human being. The Englishman's fist is more feared than the Frenchman's pistol or the dagger of the Italian. Pugilism as it is carried on is detestable; but self-defence, conducted in the good old-fashioned schoolboy style, deserves the fullest approbation.

The hard basis of the hand consists of bones—twenty-seven in number. These are divided into five different sets. The first set, commencing from the wrist-joint, is called the carpus. It has eight separate bones, all of which lie in a mass between the wrist-joint and the long straight bones of the back of the hand. The eight bones of the carpus are short and stumpy compared with the long, shaftlike bones which are interposed between them and the fingers, and which may easily be seen in outline in the hand of a very thin person.

People of a laudable degree of curiosity may ask why there should be so many bones as twenty-seven in the hand and why eight of them should, so to speak, occupy the position of mere buffers between the wrist-joint and the long bones of the back of the hand. They are, however, by no means mere buffers. Being hard bones, they impart great firmness and solidity to the hand, and being eight in number, instead of one or two, they allow a degree of mobility in the wrist joint which is quite extraordinary in so hard a structure. Strength, hardness, firmness, mobility—these are not qualities which are easily combined under any circumstances; but they are marked characteristics of the carpal portion of the human hand. Anatomists, who may be presumed to have a kind of proprietary, not to say paternal, affection for the different parts of the body, have given a separate name to each of these small bones of the carpus. The *scaphoid* is so named because it bears some resemblance to a boat. Not that the similarity is so striking as to be immediately obvious; but it may at least be said with truth that it is more like a boat than it is like a house, or a horse, or an alderman. After the scaphoid comes the *semilunar* bone—a name which explains itself. Following these are the *cuneiform* bone, which resembles a wedge or a pyramid; the *pisiform*, which is shaped like a pea; the *trapezium*, or shawl-like bone, which rejoices in the additional appellation of *os multangulum majus*; and the *trapezoid*, whose second cognomen is *os multangulum minus*. The *os magnum* is the largest bone of the carpus, as its name implies, and it is therefore further dignified with the name of *os capitatum*. Next and last is the *unciform*, or hooked bone—so-called, not because it hooks on to anything, but because it has what doctors call a "process" (a word used in this connexion because of the poverty of the anatomical imagination), and the "process" bears a very distant relationship to a hook.

The second portion of the hand, starting from the wrist-joint, is the *metacarpus*—that is, the portion beyond the carpus. This consists of five shaftlike bones, each having two heads, one at either end. The opposite extremities are here called "heads" rather than the "head" and the "foot," because they are very much alike both in shape and function. If, in order to be precise, we should call them "head" and "foot," there would be a perpetual quarrel in the scientific world as to which was the real head and which the foot. To avoid strife it is better to say that each bone of the metacarpus has two heads, one at either end. The metacarpal bones may be considered as the first five columns of the hand, standing on the foundation of the carpus. One of them connects the thumb with the carpal base, and of the other four one joins each finger with the same foundation. Muscles are attached to these bones, which, when put into action, bend the whole hand upon the forearm. An unscientific observer who sees a skeleton hand for the first time, naturally supposes that the metacarpal bones are portions of the fingers. That is not so; they, along with the carpal bones, go to the formation of the palm, and end where the fingers begin.

The proper bones of the fingers follow in order immediately after the metacarpals. They are fourteen in number, the thumb having two bones or phalanges, and the other four fingers three phalanges each. It is immediately seen, on looking at the closed fist, how much the mobility of the fingers is increased by the possession of three phalanges each rather than of two. The four digits tuck themselves into the palm with the utmost facility and neatness, and the thumb, extending over them, binds them down in the firmest possible grip. Of the three rows of phalanges which constitute the fingers, the row nearest the metacarpus has the longest bones; the next longest compose the middle phalanx, and the shortest the terminal phalanx, or finger-ends.

Fractures of the fingers are not uncommon; and if they are of the "simple" variety their management is generally easy to the experienced surgeon. Dislocations sometimes take place, and may often be righted by simply pulling the bones into position again. All kinds of injuries about the hand are more or less dangerous, on account of the wonderful number, variety, size, and importance of the blood-vessels, nerves, and muscles which belong to it. The bones of the hand, except those of the carpus, are exceedingly simple in arrangement, merely consisting of admirably-jointed rows. But for the performance of the wonderful movements of which these bones are capable, and for the supply of sensibility, motor energy, and nutrition, the nerves, blood-vessels, and muscles are marvels of complexity, completeness, and efficiency. The hand altogether is a study worthy of the profoundest attention, and if it produces no other result in the thoughtful mind, it will at least excite wonder. The conclusion which an impartial student may fitly arrive at is this, that if the human hand be the work of a conscious intelligence, it is a work of unparalleled skill; if it be the product of unintelligent and unpurposing forces, it is a mystery which no hypothesis can explain.

